# Crystal structure of ethyl 2-amino-4-(4-chloro­phen­yl)-4*H*-1-benzothieno[3,2-*b*]pyran-3-carboxyl­ate

**DOI:** 10.1107/S2056989015014085

**Published:** 2015-07-31

**Authors:** Mohamed Bakhouch, Asmae Mahfoud, Mohamed El Yazidi, Mohamed Saadi, Lahcen El Ammari

**Affiliations:** a Département de Chimie, Faculté des Sciences, Dhar Mehraz, BP 1796 Atlas, 30000 Fes, Morocco; b Laboratoire de Chimie du Solide Appliquée, Faculté des Sciences, Université Mohammed V, Avenue Ibn Battouta, BP 1014, Rabat, Morocco

**Keywords:** crystal structure, thio­aurones, thio­phenones, benzothieno­pyran, N—H⋯O hydrogen bonds, inversion dimers, Cl⋯O short contact.

## Abstract

The title compound, C_20_H_16_ClNO_3_S, is built up from three fused rings, one five- and two six-membered rings, linked to a 3-eth­oxy­carbonyl group and to a 4-chloro­phenyl ring. The hydropyran ring has a flattened envelope conformation, with the C atom substituted by the 4-chloro­phenyl ring as the flap (displaced by 0.077 (2) Å from the plane through the other atoms). The fused three-ring system is quasi-planar (r.m.s. deviation = 0.057 Å), with the largest deviation from the mean plane being 0.106 (1) Å for the C atom substituted by the 4-chloro­phenyl ring. The 4-chloro­phenyl ring is approximately perpendicular to the mean plane of the fused ring system, as indicated by the dihedral angle of 77.32 (6)° between their mean planes. There is an intra­molecular N—H⋯O hydrogen bond forming an *S*(6) ring motif. In the crystal, mol­ecules are linked by pairs of N—H⋯O hydrogen bonds, forming inversion dimers with an *R*_2_^2^(12) ring motif. There are also short inter­molecular Cl⋯O inter­actions present [3.1226 (12) Å] between neighbouring mol­ecules.

## Related literature   

For the reactivity of the thio­aurones [(*Z*)-2-aryl­idene­benzo[*b*]thio­phen-3(2*H*)-ones], see: Boughaleb *et al.* (2010 ▸, 2011 ▸); Bakhouch *et al.* (2014 ▸, 2015 ▸); Cabiddu *et al.* (2002 ▸); Pradhan *et al.* (2005 ▸). For the preparation of the title compound using condensation reactions, see: Daisley *et al.* (1982 ▸).
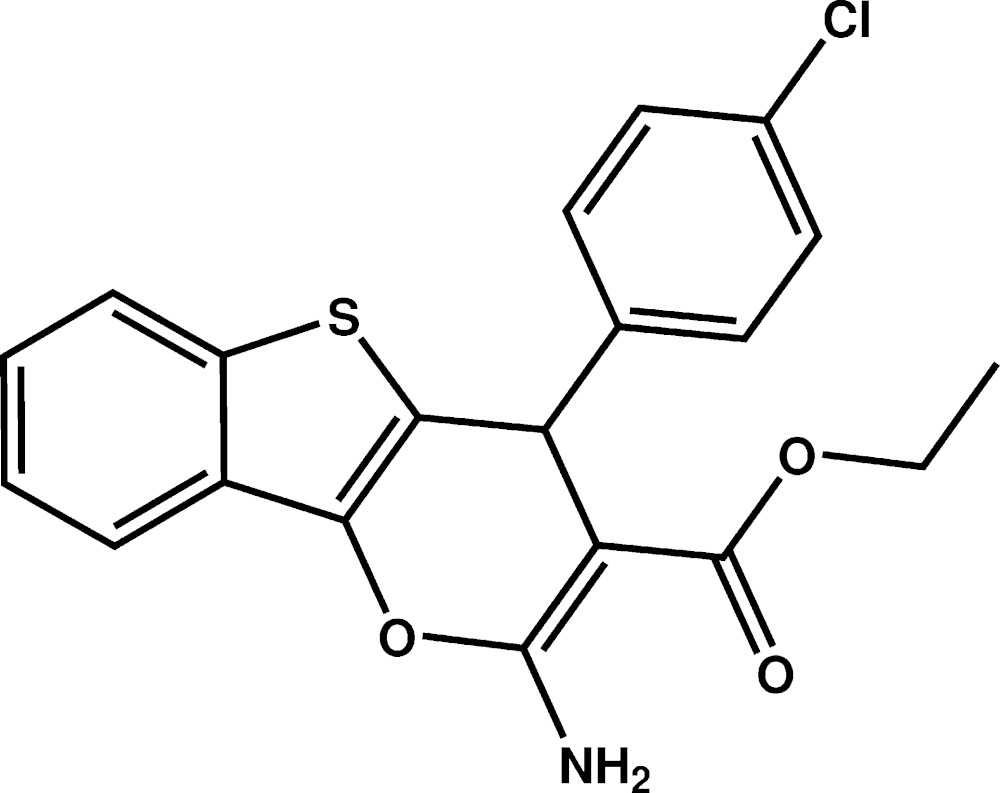


## Experimental   

### Crystal data  

C_20_H_16_ClNO_3_S
*M* *_r_* = 385.85
Triclinic, 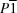

*a* = 8.3606 (4) Å
*b* = 10.9186 (6) Å
*c* = 11.0971 (6) Å
α = 104.592 (2)°
β = 106.849 (2)°
γ = 102.174 (2)°
*V* = 893.09 (8) Å^3^
*Z* = 2
Mo *K*α radiation
μ = 0.35 mm^−1^
*T* = 296 K
0.42 × 0.31 × 0.26 mm

### Data collection  

Bruker X8 APEX diffractometer
Absorption correction: multi-scan (*SADABS*; Bruker, 2009 ▸) *T*_min_ = 0.673, *T*_max_ = 0.746
22918 measured reflections
3892 independent reflections
3422 reflections with *I* > 2σ(*I*)
*R*_int_ = 0.029

### Refinement  

*R*[*F* ^2^ > 2σ(*F* ^2^)] = 0.033
*wR*(*F* ^2^) = 0.097
*S* = 1.04
3892 reflections
235 parameters
H-atom parameters constrained
Δρ_max_ = 0.30 e Å^−3^
Δρ_min_ = −0.24 e Å^−3^

### 

Data collection: *APEX2* (Bruker, 2009 ▸); cell refinement: *SAINT* (Bruker, 2009 ▸); data reduction: *SAINT*; program(s) used to solve structure: *SHELXS2013* (Sheldrick, 2008 ▸); program(s) used to refine structure: *SHELXL2013* (Sheldrick, 2015 ▸); molecular graphics: *ORTEP-3 for Windows* (Farrugia, 2012 ▸) and *PLATON* (Spek, 2009 ▸); software used to prepare material for publication: *SHELXL2013*, *PLATON* and *publCIF* (Westrip, 2010 ▸).

## Supplementary Material

Crystal structure: contains datablock(s) I. DOI: 10.1107/S2056989015014085/su5182sup1.cif

Structure factors: contains datablock(s) I. DOI: 10.1107/S2056989015014085/su5182Isup2.hkl

Supporting information file. DOI: 10.1107/S2056989015014085/su5182Isup3.cml

**Fig. 1.**
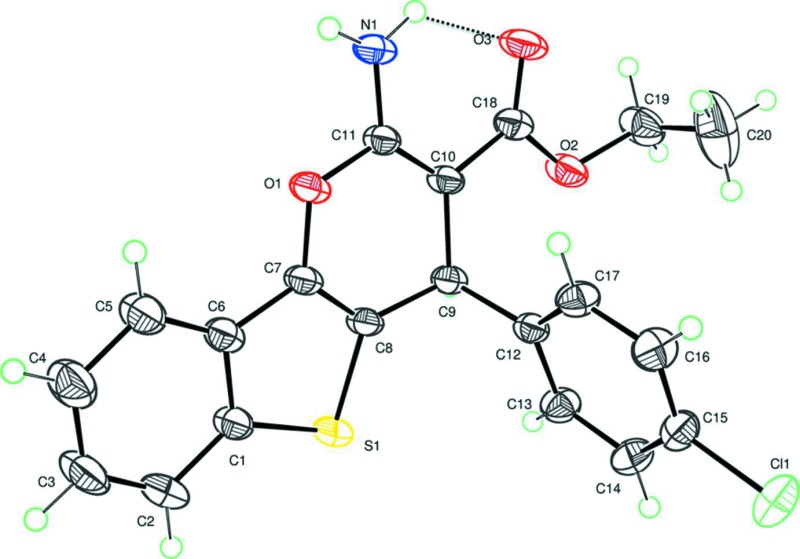
A view of the molecule structure of the title compound, with the atom labelling. Displacement ellipsoids are drawn at the 50% probability level. The intramolecular N-H···O hydrogen bond is shown as a dashed line (see Table 1).

**Fig. 2.**
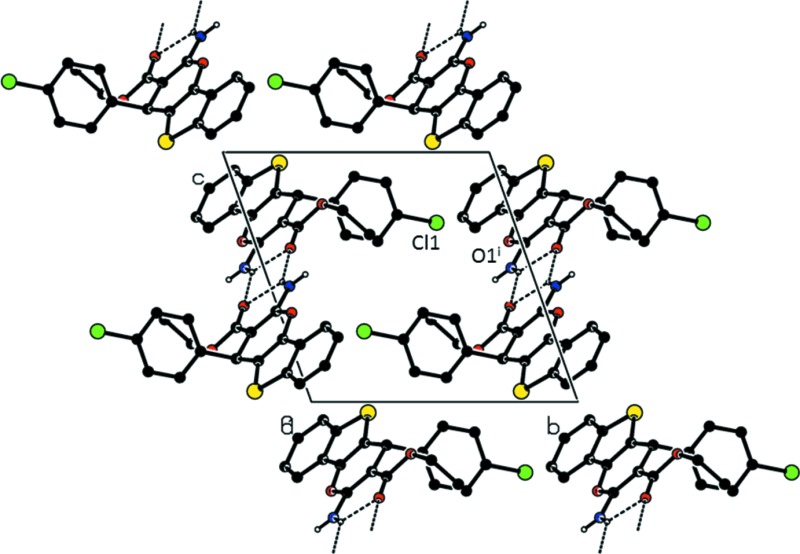
A view along the a axis of the crystal packing for the title compound. The hydrogen bonds are shown as dashed lines (see Table 1), and C-bound H atoms have been omitted for clarity.

. DOI: 10.1107/S2056989015014085/su5182fig1.tif
A view of the mol­ecule structure of the title compound, with the atom labelling. Displacement ellipsoids are drawn at the 50% probability level. The intra­molecular N-H⋯O hydrogen bond is shown as a dashed line (see Table 1).

a . DOI: 10.1107/S2056989015014085/su5182fig2.tif
A view along the *a* axis of the crystal packing for the title compound. The hydrogen bonds are shown as dashed lines (see Table 1), and C-bound H atoms have been omitted for clarity.

CCDC reference: 1415291

Additional supporting information:  crystallographic information; 3D view; checkCIF report

## Figures and Tables

**Table 1:** Hydrogen-bond geometry (, )

*D*H*A*	*D*H	H*A*	*D* *A*	*D*H*A*
N1H1*A*O3	0.86	2.07	2.6796(18)	127
N1H1*A*O3^i^	0.86	2.18	2.8956(15)	141

Symmetry code: (i) 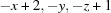.

## supplementary crystallographic information

### S1. Comments

During our studies on the synthesis of heterocyclic compounds (Boughaleb *et al.*, 2010,2011), we decided to investigate thio­aurones [(*Z*)-2-aryl­idenebenzo[*b*]thio­phen-3(2*H*)-ones] as potential starting materials (Cabiddu *et al.*, 2002; Pradhan & Asish, 2005). In continuation of our previous work (Boughaleb *et al.*, 2011; Bakhouch *et al.*, 2014), we described herein the behaviour of ethyl cyano­acetate with (*Z*)-2-(4-chloro­benzyl­idene)benzo[*b*]thio­phen-3(2*H*)-one. The title compound, was prepared by the action of ethyl cyano­acetate on (*Z*)-2-(4-chloro­benzyl­idene)benzo[*b*]thio­phen-3(2*H*)-one. The reaction was carried out in hot alcohol in the presence of piperidine as a basic catalyst (Daisley *et al.*, 1982). Initially the condensation gave the Michael adducts which undergoes intra­molecular cyclization to afford an imino-pyran. The subsequent tautomeric transformation gives rise to the title compound, whose crystal structure we report on herein.

The molecule of the title compound, Fig. 1, is formed by three fused rings linked to an ethyl-3-carboxyl­ate group and to a 4-chloro­phenyl. The three fused rings (S1/C1—C11/O1) are nearly coplanar, with the maximum deviation from the mean plane being -0.106 (1) Å for atom C9. Its mean plane make a dihedral angle of 77.32 (6)° with the attached 4-chloro­phenyl ring. The pyran ring has a flat envelope conformation with atom C9, substituted by the 4-chloro­phenyl ring, as the flap. There is an intra­molecular N—H···O hydrogen bond, involving the amine and carboxyl ate group, forming an S(6) ring motif (Fig. 1 and Table 1).

In the crystal, molecules are linked by pairs of N—H···O hydrogen bonds forming inversion dimers with an *R*_2_^2^(12) ring motif (Table 1 and Fig. 2). There are also short inter­molecular Cl1···O1^i^ inter­actions present between neighbouring molecules [3.1226 (12) Å; symmetry code: (i) x, y+1, z; see Fig. 2].

### S2. Synthesis and crystallization

In a 100 ml flask equipped with a condenser was dissolved 4 mmol of (*Z*)-2-(4-chloro­benzyl­idene)-1-benzo[*b*]thio­phen-3(2*H*)-one and 5 mmol of ethyl cyano­acetate in 30 ml of ethanol. Then, 1 ml of piperidine was added, and the reaction mixture was refluxed for 6 h. Thin layer chromatography revealed the formation of a single product. The organic phase was evaporated under reduce pressure. The resulting residue was recrystallized from ethanol (yield: 67%; m.p.: 405 K). Colourless block-like crystals of the title compound were obtained by slow evaporation of a solution in ethanol.

### S3. Refinement

Crystal data, data collection and structure refinement details are summarized in Table 2. H atoms were located in a difference map and treated as riding: N—H = 0.86 Å, C–H = 0.93 - 0.98Å with *U*_iso_(H) = 1.5*U*_eq_(C) for methyl H atoms and 1.2*U*_eq_(N,C) for other H atoms.

### Crystal data

**Table:**

C_20_H_16_ClNO_3_S	*Z* = 2
*M**_r_* = 385.85	*F*(000) = 400
Triclinic, *P*1	*D*_x_ = 1.435 Mg m^−^^3^
*a* = 8.3606 (4) Å	Mo *K*α radiation, λ = 0.71073 Å
*b* = 10.9186 (6) Å	Cell parameters from 3892 reflections
*c* = 11.0971 (6) Å	θ = 2.3–27.0°
α = 104.592 (2)°	µ = 0.35 mm^−^^1^
β = 106.849 (2)°	*T* = 296 K
γ = 102.174 (2)°	Block, colourless
*V* = 893.09 (8) Å^3^	0.42 × 0.31 × 0.26 mm

### Data collection

**Table:**

Bruker X8 APEX diffractometer	3892 independent reflections
Radiation source: fine-focus sealed tube	3422 reflections with *I* > 2σ(*I*)
Graphite monochromator	*R*_int_ = 0.029
φ and ω scans	θ_max_ = 27.0°, θ_min_ = 2.3°
Absorption correction: multi-scan (*SADABS*; Bruker, 2009)	*h* = −10→10
*T*_min_ = 0.673, *T*_max_ = 0.746	*k* = −13→13
22918 measured reflections	*l* = −14→14

### Refinement

**Table:**

Refinement on *F*^2^	0 restraints
Least-squares matrix: full	Hydrogen site location: inferred from neighbouring sites
*R*[*F*^2^ > 2σ(*F*^2^)] = 0.033	H-atom parameters constrained
*wR*(*F*^2^) = 0.097	*w* = 1/[σ^2^(*F*_o_^2^) + (0.0548*P*)^2^ + 0.2464*P*] where *P* = (*F*_o_^2^ + 2*F*_c_^2^)/3
*S* = 1.04	(Δ/σ)_max_ = 0.001
3892 reflections	Δρ_max_ = 0.30 e Å^−^^3^
235 parameters	Δρ_min_ = −0.24 e Å^−^^3^

### Special details

**Table:**

Geometry. All e.s.d.'s (except the e.s.d. in the dihedral angle between two l.s. planes) are estimated using the full covariance matrix. The cell e.s.d.'s are taken into account individually in the estimation of e.s.d.'s in distances, angles and torsion angles; correlations between e.s.d.'s in cell parameters are only used when they are defined by crystal symmetry. An approximate (isotropic) treatment of cell e.s.d.'s is used for estimating e.s.d.'s involving l.s. planes.

### Fractional atomic coordinates and isotropic or equivalent isotropic displacement parameters (Å^2^)

**Table:**

	*x*	*y*	*z*	*U*_iso_*/*U*_eq_	
C1	0.35626 (19)	0.05325 (15)	0.88366 (15)	0.0351 (3)	
C2	0.2174 (2)	0.01998 (18)	0.92720 (18)	0.0471 (4)	
H2	0.2128	0.0769	1.0033	0.057*	
C3	0.0882 (2)	−0.0988 (2)	0.85487 (19)	0.0542 (4)	
H3	−0.0054	−0.1223	0.8824	0.065*	
C4	0.0936 (2)	−0.1852 (2)	0.74085 (19)	0.0539 (4)	
H4	0.0034	−0.2648	0.6931	0.065*	
C5	0.2317 (2)	−0.15375 (17)	0.69832 (16)	0.0432 (4)	
H5	0.2359	−0.2120	0.6229	0.052*	
C6	0.36455 (18)	−0.03341 (14)	0.77004 (14)	0.0325 (3)	
C7	0.51995 (18)	0.02422 (13)	0.74848 (13)	0.0296 (3)	
C8	0.62124 (18)	0.14449 (13)	0.83590 (13)	0.0294 (3)	
C9	0.78415 (17)	0.22422 (13)	0.82693 (13)	0.0281 (3)	
H9	0.8804	0.2515	0.9131	0.034*	
C10	0.82868 (17)	0.13392 (13)	0.72085 (13)	0.0298 (3)	
C11	0.71717 (18)	0.01172 (13)	0.63660 (13)	0.0298 (3)	
C12	0.75379 (16)	0.34879 (13)	0.79785 (13)	0.0274 (3)	
C13	0.7782 (2)	0.46098 (14)	0.90231 (14)	0.0354 (3)	
H13	0.8217	0.4617	0.9898	0.042*	
C14	0.7389 (2)	0.57212 (14)	0.87879 (15)	0.0385 (3)	
H14	0.7540	0.6462	0.9494	0.046*	
C15	0.67728 (18)	0.57080 (13)	0.74900 (15)	0.0346 (3)	
C16	0.6569 (2)	0.46233 (15)	0.64347 (15)	0.0396 (3)	
H16	0.6191	0.4637	0.5564	0.047*	
C17	0.69348 (19)	0.35146 (14)	0.66880 (14)	0.0349 (3)	
H17	0.6772	0.2774	0.5977	0.042*	
C18	0.99172 (18)	0.18273 (14)	0.70204 (14)	0.0338 (3)	
C19	1.2395 (2)	0.37234 (19)	0.76685 (18)	0.0508 (4)	
H19A	1.2933	0.3086	0.7312	0.061*	
H19B	1.3263	0.4365	0.8501	0.061*	
C20	1.1832 (4)	0.4415 (3)	0.6705 (3)	0.1006 (10)	
H20A	1.2833	0.4864	0.6551	0.151*	
H20B	1.1310	0.5052	0.7063	0.151*	
H20C	1.0989	0.3777	0.5877	0.151*	
N1	0.74061 (17)	−0.07170 (12)	0.53678 (13)	0.0404 (3)	
H1A	0.8348	−0.0502	0.5199	0.048*	
H1B	0.6615	−0.1469	0.4894	0.048*	
O1	0.55816 (13)	−0.04447 (10)	0.64299 (10)	0.0341 (2)	
O2	1.09024 (13)	0.30414 (11)	0.79199 (11)	0.0412 (3)	
O3	1.03951 (15)	0.12634 (12)	0.61632 (12)	0.0503 (3)	
S1	0.53387 (5)	0.19821 (4)	0.95704 (4)	0.03808 (12)	
Cl1	0.62208 (6)	0.70800 (4)	0.71743 (4)	0.05029 (13)	

### Atomic displacement parameters (Å^2^)

**Table:**

	*U*^11^	*U*^22^	*U*^33^	*U*^12^	*U*^13^	*U*^23^
C1	0.0365 (7)	0.0397 (7)	0.0395 (7)	0.0175 (6)	0.0224 (6)	0.0158 (6)
C2	0.0454 (9)	0.0575 (10)	0.0523 (9)	0.0213 (8)	0.0346 (8)	0.0170 (8)
C3	0.0390 (9)	0.0698 (12)	0.0624 (11)	0.0136 (8)	0.0341 (8)	0.0206 (9)
C4	0.0365 (8)	0.0595 (11)	0.0591 (11)	0.0042 (8)	0.0223 (8)	0.0119 (9)
C5	0.0378 (8)	0.0492 (9)	0.0420 (8)	0.0101 (7)	0.0203 (7)	0.0099 (7)
C6	0.0316 (7)	0.0393 (7)	0.0351 (7)	0.0159 (6)	0.0179 (6)	0.0158 (6)
C7	0.0324 (7)	0.0354 (7)	0.0311 (6)	0.0175 (5)	0.0190 (5)	0.0131 (5)
C8	0.0348 (7)	0.0329 (7)	0.0307 (6)	0.0173 (5)	0.0202 (5)	0.0121 (5)
C9	0.0306 (6)	0.0313 (6)	0.0270 (6)	0.0136 (5)	0.0150 (5)	0.0087 (5)
C10	0.0312 (7)	0.0333 (7)	0.0335 (7)	0.0163 (5)	0.0192 (5)	0.0116 (5)
C11	0.0336 (7)	0.0330 (7)	0.0331 (6)	0.0172 (5)	0.0198 (5)	0.0133 (5)
C12	0.0250 (6)	0.0296 (6)	0.0292 (6)	0.0092 (5)	0.0134 (5)	0.0076 (5)
C13	0.0428 (8)	0.0348 (7)	0.0273 (6)	0.0137 (6)	0.0131 (6)	0.0063 (5)
C14	0.0444 (8)	0.0291 (7)	0.0364 (7)	0.0118 (6)	0.0135 (6)	0.0023 (6)
C15	0.0305 (7)	0.0283 (7)	0.0430 (8)	0.0097 (5)	0.0104 (6)	0.0113 (6)
C16	0.0444 (8)	0.0427 (8)	0.0309 (7)	0.0170 (7)	0.0100 (6)	0.0123 (6)
C17	0.0405 (8)	0.0347 (7)	0.0280 (6)	0.0148 (6)	0.0122 (6)	0.0053 (5)
C18	0.0327 (7)	0.0387 (7)	0.0360 (7)	0.0155 (6)	0.0183 (6)	0.0115 (6)
C19	0.0330 (8)	0.0570 (10)	0.0541 (10)	0.0021 (7)	0.0224 (7)	0.0070 (8)
C20	0.0660 (15)	0.115 (2)	0.130 (2)	0.0027 (15)	0.0361 (16)	0.078 (2)
N1	0.0442 (7)	0.0356 (6)	0.0458 (7)	0.0121 (5)	0.0308 (6)	0.0040 (5)
O1	0.0347 (5)	0.0343 (5)	0.0358 (5)	0.0099 (4)	0.0222 (4)	0.0054 (4)
O2	0.0327 (5)	0.0451 (6)	0.0417 (6)	0.0059 (4)	0.0211 (5)	0.0039 (5)
O3	0.0465 (6)	0.0504 (7)	0.0569 (7)	0.0128 (5)	0.0373 (6)	0.0029 (5)
S1	0.0447 (2)	0.0385 (2)	0.0392 (2)	0.01531 (16)	0.02879 (17)	0.00859 (15)
Cl1	0.0518 (2)	0.0325 (2)	0.0608 (3)	0.01558 (17)	0.00947 (19)	0.01663 (18)

### Geometric parameters (Å, º)

**Table:**

C1—C2	1.396 (2)	C12—C17	1.3837 (19)
C1—C6	1.4025 (19)	C12—C13	1.3885 (18)
C1—S1	1.7412 (16)	C13—C14	1.388 (2)
C2—C3	1.369 (3)	C13—H13	0.9300
C2—H2	0.9300	C14—C15	1.377 (2)
C3—C4	1.394 (3)	C14—H14	0.9300
C3—H3	0.9300	C15—C16	1.380 (2)
C4—C5	1.379 (2)	C15—Cl1	1.7441 (14)
C4—H4	0.9300	C16—C17	1.384 (2)
C5—C6	1.393 (2)	C16—H16	0.9300
C5—H5	0.9300	C17—H17	0.9300
C6—C7	1.4327 (18)	C18—O3	1.2134 (17)
C7—C8	1.340 (2)	C18—O2	1.3521 (18)
C7—O1	1.3821 (15)	C19—O2	1.4507 (18)
C8—C9	1.4983 (18)	C19—C20	1.484 (3)
C8—S1	1.7428 (13)	C19—H19A	0.9700
C9—C10	1.5235 (16)	C19—H19B	0.9700
C9—C12	1.5289 (17)	C20—H20A	0.9600
C9—H9	0.9800	C20—H20B	0.9600
C10—C11	1.366 (2)	C20—H20C	0.9600
C10—C18	1.4492 (19)	N1—H1A	0.8600
C11—N1	1.3371 (17)	N1—H1B	0.8600
C11—O1	1.3717 (16)		
			
C2—C1—C6	120.93 (15)	C13—C12—C9	119.77 (12)
C2—C1—S1	126.81 (13)	C14—C13—C12	121.31 (13)
C6—C1—S1	112.26 (10)	C14—C13—H13	119.3
C3—C2—C1	118.18 (15)	C12—C13—H13	119.3
C3—C2—H2	120.9	C15—C14—C13	118.84 (13)
C1—C2—H2	120.9	C15—C14—H14	120.6
C2—C3—C4	121.53 (15)	C13—C14—H14	120.6
C2—C3—H3	119.2	C14—C15—C16	121.13 (13)
C4—C3—H3	119.2	C14—C15—Cl1	119.42 (11)
C5—C4—C3	120.57 (17)	C16—C15—Cl1	119.45 (11)
C5—C4—H4	119.7	C15—C16—C17	119.13 (13)
C3—C4—H4	119.7	C15—C16—H16	120.4
C4—C5—C6	118.99 (15)	C17—C16—H16	120.4
C4—C5—H5	120.5	C12—C17—C16	121.25 (13)
C6—C5—H5	120.5	C12—C17—H17	119.4
C5—C6—C1	119.78 (13)	C16—C17—H17	119.4
C5—C6—C7	130.77 (13)	O3—C18—O2	121.50 (13)
C1—C6—C7	109.45 (13)	O3—C18—C10	126.35 (14)
C8—C7—O1	124.03 (12)	O2—C18—C10	112.14 (11)
C8—C7—C6	115.98 (12)	O2—C19—C20	110.42 (16)
O1—C7—C6	119.99 (12)	O2—C19—H19A	109.6
C7—C8—C9	124.22 (11)	C20—C19—H19A	109.6
C7—C8—S1	111.10 (10)	O2—C19—H19B	109.6
C9—C8—S1	124.64 (10)	C20—C19—H19B	109.6
C8—C9—C10	107.59 (11)	H19A—C19—H19B	108.1
C8—C9—C12	109.14 (10)	C19—C20—H20A	109.5
C10—C9—C12	113.90 (10)	C19—C20—H20B	109.5
C8—C9—H9	108.7	H20A—C20—H20B	109.5
C10—C9—H9	108.7	C19—C20—H20C	109.5
C12—C9—H9	108.7	H20A—C20—H20C	109.5
C11—C10—C18	118.03 (12)	H20B—C20—H20C	109.5
C11—C10—C9	123.05 (11)	C11—N1—H1A	120.0
C18—C10—C9	118.79 (12)	C11—N1—H1B	120.0
N1—C11—C10	127.48 (13)	H1A—N1—H1B	120.0
N1—C11—O1	108.97 (12)	C11—O1—C7	116.36 (11)
C10—C11—O1	123.55 (11)	C18—O2—C19	116.32 (11)
C17—C12—C13	118.29 (12)	C1—S1—C8	91.21 (7)
C17—C12—C9	121.86 (11)		

### Hydrogen-bond geometry (Å, º)

**Table:**

*D*—H···*A*	*D*—H	H···*A*	*D*···*A*	*D*—H···*A*
N1—H1*A*···O3	0.86	2.07	2.6796 (18)	127
N1—H1*A*···O3^i^	0.86	2.18	2.8956 (15)	141

Symmetry code: (i) −*x*+2, −*y*, −*z*+1.
