# Supplementary figures and images for: Crystal structure of ethyl 2-amino-4-(4-chloro­phen­yl)-4H-1-benzothieno[3,2-b]pyran-3-carboxyl­ate
Source: Acta Crystallogr E Crystallogr Commun. 2015 Jul 31;71(Pt 8):o619–20. doi: 10.1107/S2056989015014085 (PMC4571428; doi:10.1107/S2056989015014085)

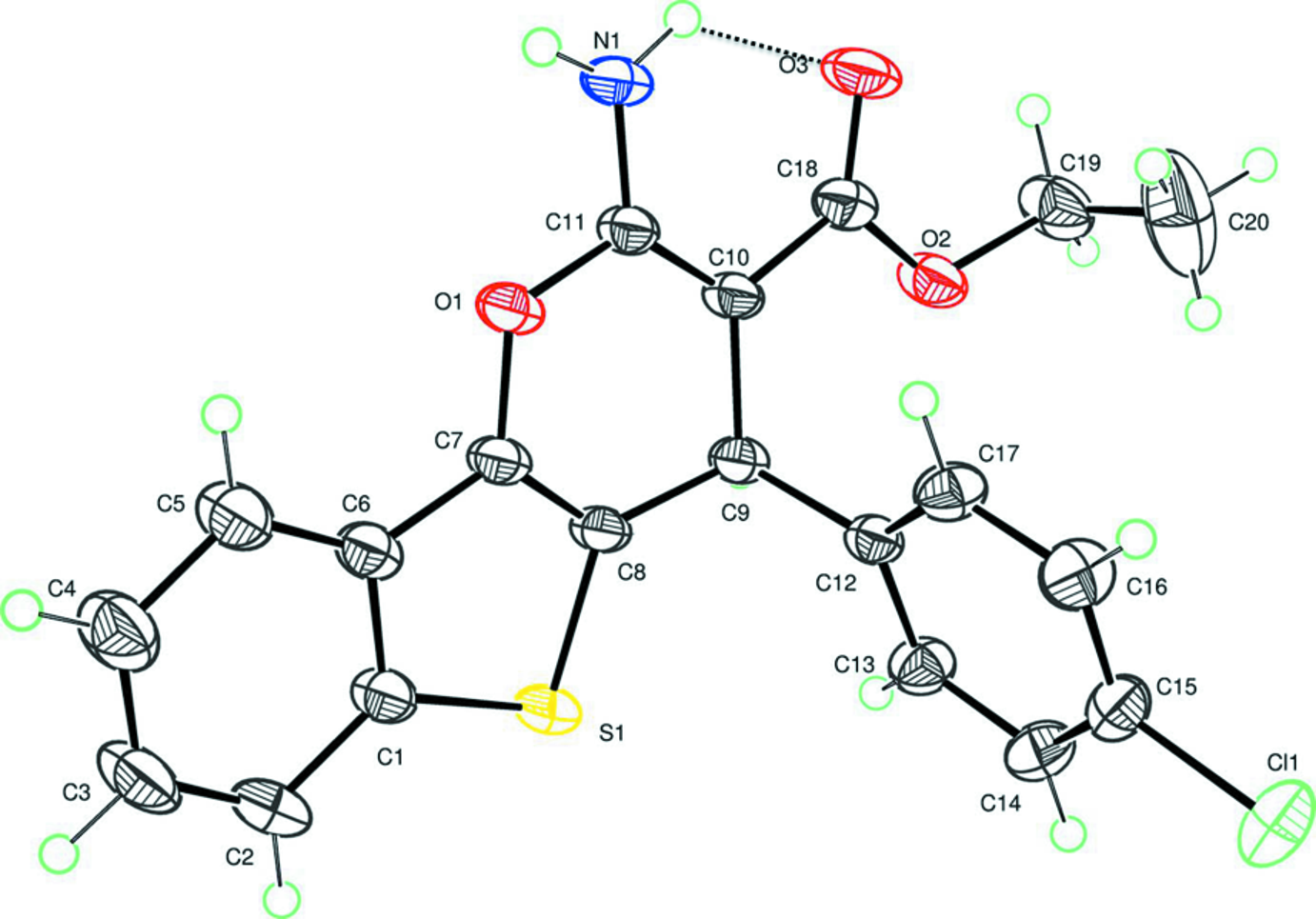

Supplement: Supplementary file 4 [file e-71-0o619-fig1.tif]

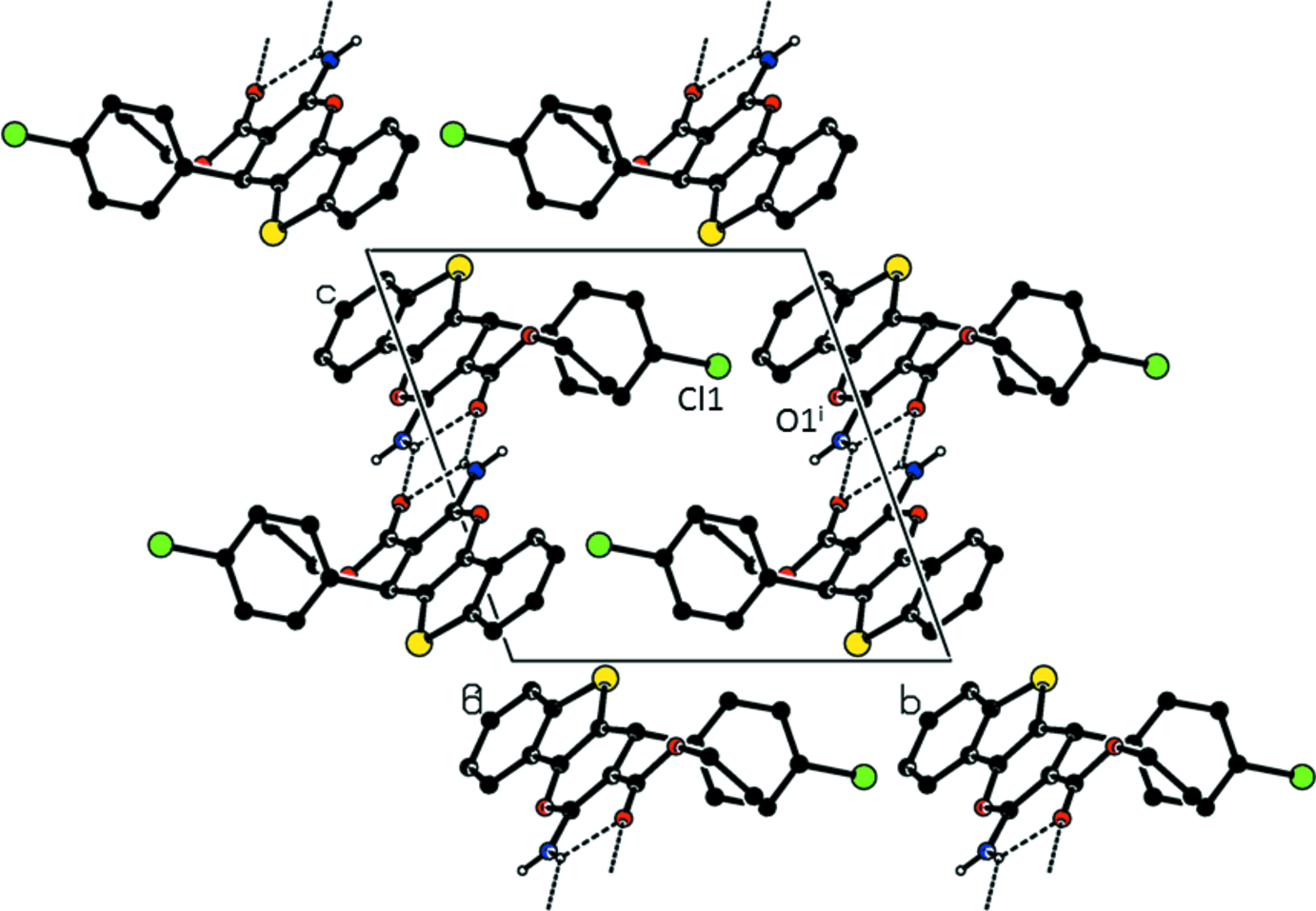

Supplement: Supplementary file 5 [file e-71-0o619-fig2.tif]
